# Elevator Cabin Decontamination With ACTIVE Particle Control™ Technology

**DOI:** 10.3389/fpubh.2021.729204

**Published:** 2021-12-08

**Authors:** Mark Ereth, Tracy Wagoner, Mark Blevins, Donald Hess

**Affiliations:** ^1^Mayo Clinic, Rochester, MN, United States; ^2^Henderson Building Solutions, LLC, Lenexa, KS, United States; ^3^SecureAire, Inc., Dunedin, FL, United States

**Keywords:** aerosol disinfection, building elevators, particle count, viral disease transmission, indoor air quality

## Abstract

Effectively reducing contamination and aerosolized bioburden may limit the risk of disease transmission in closed settings when social distancing is not possible. Unlike uncontrolled ionization and oxidation devices ACTIVE Particle Control™ conditions particles in a highly controlled fashion which provides effective air purification without the generation of ozone or other toxic by-products. The purpose of this study was to determine the impact of ACTIVE Particle Control™ on elevator cabin particle load compared to standard ventilation. The intervention trial utilized particle mass tools to determine the difference in particle clearance between standard elevator cabin ventilation and ACTIVE Particle Control™ technology. Cabin particulate contaminants were significantly reduced using ACTIVE Particle Control™ technology in an operating elevator.

## Introduction

Indoor air bioaerosols that carry bacteria, viruses, and fungi serve as a transmission vehicle for diverse infections, including influenza viruses, severe acute respiratory syndrome viruses, and the novel human coronavirus (SARS-CoV-2). Elevator cabins have fans, filters, and/or vents to facilitate air flow but provide limited fresh air and generally do not have air purification technology capable of clearing particulate or pathogen contamination (1–3). Pathogen droplets and aerosols can persist in operating hospital elevator cabins for up to 18 min (4). New passengers may inhale the exhaled aerosols from other passengers who rode that car during the previous 18–25 min (4). The relative risk of viral transmission during a brief elevator ride is less than an hour in a crowded commuter bus but is not insignificant (1). The SARS-CoV-2 virus can remain viable for up to 3 h after aerosolization and the pathogen payload from an infected fellow passenger can be carried much further than the commonly accepted 6 feet of social distancing (5, 6). Disinfection of elevator cabin air could significantly reduce the potential for airborne transmission of disease and real-time monitoring of non-viable airborne particles has been shown to correlate with airborne pathogens (7). ACTIVE Particle Control™ (APC) technology (SecureAire, Inc., Dunedin, FL) has been shown to reduce fine and ultrafine airborne particles and pathogens in live operating rooms, reduce bacterial contamination in active hospital compounding pharmacies, rapidly inactivate or kill the highly resistant Anthrax surrogate (*Bacillus subtilis*) in a research laboratory (8). APC has also reduced health care-associated infections by 45% (9). This novel technology works by local electromagnetic field manipulation (controlled ionization and polarization) (10, 11). These forces condition the microparticles and pathogens within a space, so they continuously initiate millions of particle-particle (ionization) and particle-molecular (polarization) collisions. These collisions lead to immediate and permanent ionically driven aggregations of fine and ultrafine particles and pathogens into larger particles. With the larger aggregates attaining a critical mass, they fall under the greater influence of physical forces and can be carried by air currents to the particle collector, and/or driven out of the occupied space *via* an exhaust pathway. The behavior of all particles (which include aerosolized or airborne viruses and bacteria) is guided principally by the physical laws of science, and to a much smaller degree, chemical and biological influences (12). It's these mass and spatial characteristics of these particles (which include airborne virus and bacteria) that keep them suspended for long periods of time allowing for wide field dispersion or diffusion (13). Upon aggregation, particles and pathogens build mass which alters their subjectivity to the physical laws of science. Specifically, the increased mass results in the reduction of influence by electrostatic forces and increases their sensitivity to physical forces such as air currents and gravity.

While some of the largest particles experienced decreased settling times and begin to drop, the majority of these larger particles are swept away by air currents to the particle collector or exhaust. The aggregated particles trapped at the collector are then subjected to a high-voltage electric field that inactivates or kills the previously airborne viruses, bacteria, and spores *via* oxidative stress.

Unlike most ionizers which impart a single charge (positive or negative) to airborne particles, the mechanism of APC imparts both positive and negative charges, in a controlled manner utilizing Gausses Law (Maxwell's first equation), on individual particles and pathogens. This is completed *via* the control mechanism which manipulates (*via* the device hardware, firmware, and software) the electronic forces applied to the sub- and supra-micron sized particles. As the agglomerated particles exit the activator system net-neutral particles are created which recruit others from the ventilated space. This distinguishes itself from bipolar ionization which emits positive and/or negative ions in an uncontrolled manner which can create detrimental entities such as ozone. By placing both positive and negative charges (in a controlled manner) on individual particles, these net-neutral particles themselves are attracted to each other rather being attracted to surfaces, valuable objects, and pets or humans in the occupied space. The authors hypothesized that APC would effectively decontaminate elevator cabins as measured by airborne microparticle counts.

## Methods

**Figure 1:**
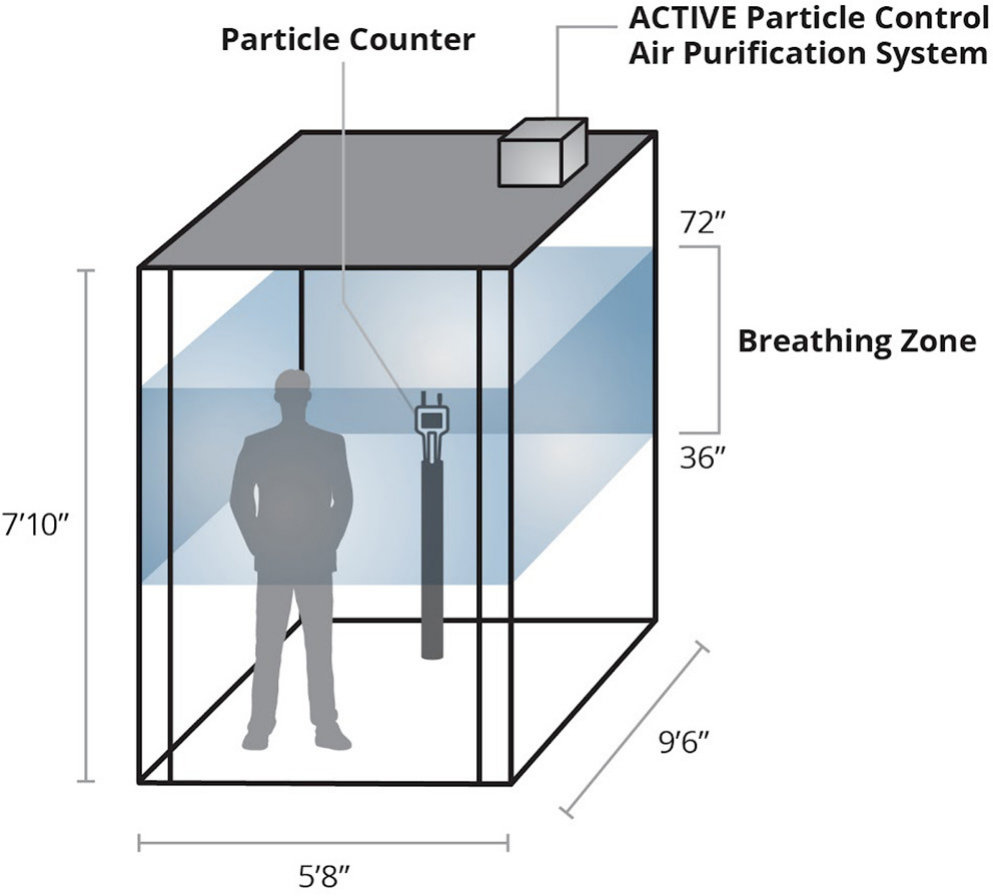
Experimental schematic including placement of indoor air particle counter and ACTIVE Particle Control™ unit.

A Dover DMC-1 hydraulic elevator cabin that served four floors in a mid-sized office building was studied. This elevator was the middle of three cars in the hoist-way and measured 9'6”L ×5'8”W ×7'9.5”H (414 ft^3^) (Figure 1). The control ventilation consisted of a standard elevator fan operating on medium speed with a minimum efficiency reporting value (MERV) 13 rating providing 27 air changes per hour to the cabin. The air quality monitor sampled cabin air at 42” above the floor when mounted on a small stand at left rear of the elevator cabin. The SecureAire unit replaced the standard (exisiting) fan/filter unit at the right rear roof of the elevator cabin. A ridership study was conducted on this elevator cabin prior to the particle counting component.

Control particle mass was obtained in this operating elevator cabin with a GrayWolf PC-3016A particle mass counter and a GrayWolf IAQ probe (GrayWolf Sensing Solutions, Shelton, CT). The sampling port was at or just below the typical passenger breathing zone. The total particle mass (ug/m^3^) of particulate matter (PM); PM 0.3 micron, PM 0.5 micron, PM 1.0 micron, PM 2.5 microns, PM 5.0 microns, and PM 10.0 microns were each obtained for 100 consecutive minutes during late morning operation for each of the control and intervention periods. An APC device with a MERV 15 rating was then installed in place of the elevator ceiling fan above the cabin ceiling and the intervention measurements were collected. A 100 min segment was chosen as a sizable sample for mid-morning average traffic as opposed to the more variable early morning, mid-day, or later afternoon elevator traffic. The mean, standard deviation, median, and interquartile ranges were determined for each PM size and mass. Comparisons were made between the baseline and treatment intervals with a parametric paired *T*-test for change and a non-parametric *T*-test for change.

## Results and Discussion

During the experiment the temperature was maintained between 23.7 and 25.3°C and humidity ranged between 40.7 and 45.7%. The results for the days of the week and time intervals for the particle count were an average of 20 single riders each hour. The ridership remained consistent throughout the particle count study.

**Figure 2:**
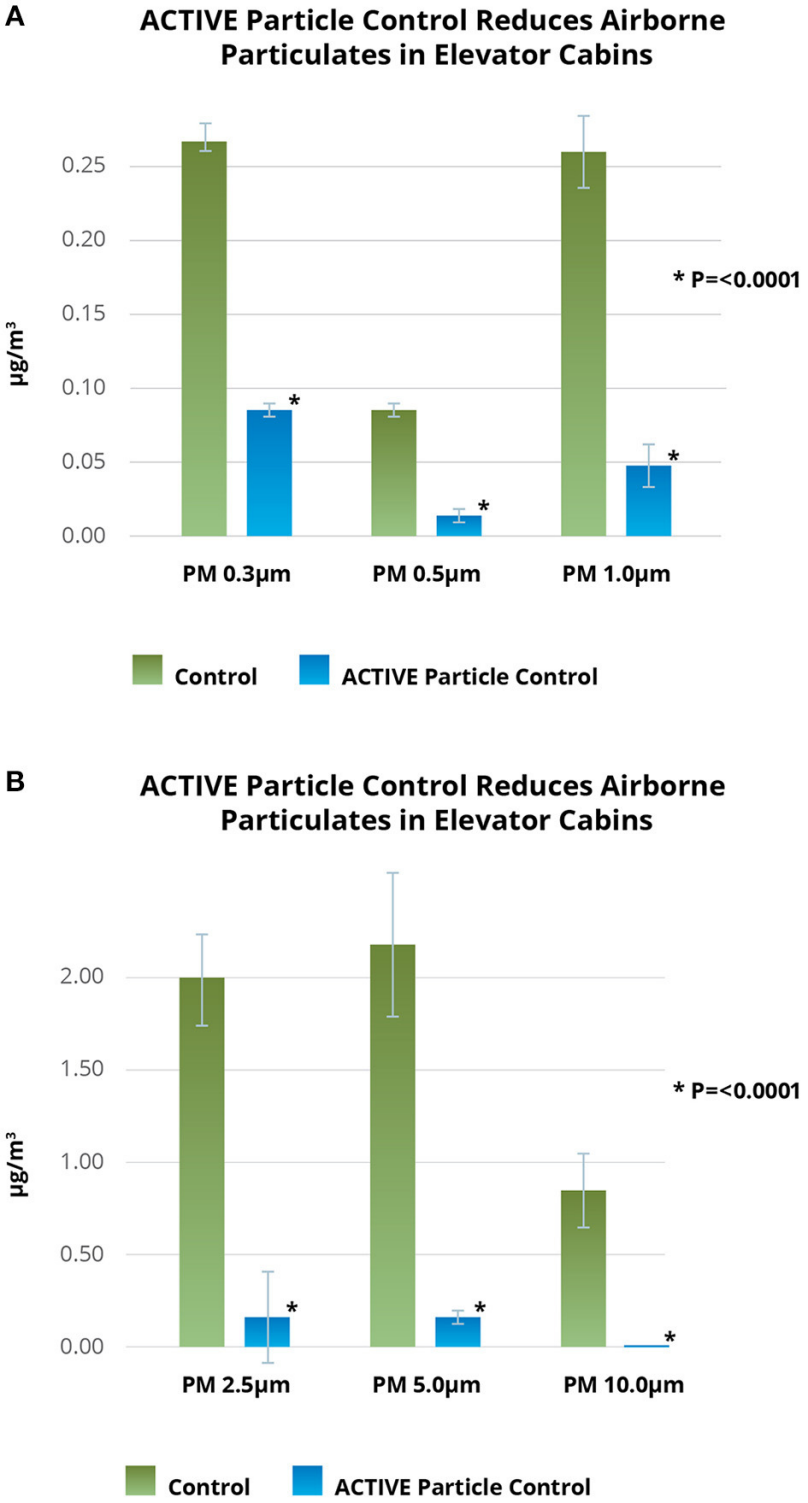
**(A,B)** ACTIVE Particle Control™ decontaminates elevator cabin air. ACTIVE Particle Control^TM^ reduced sub-micron and supra-micron particles (ug/m^3^) by 77-100% (mean 88%; *p* <0.001) in an active elevator cabin. Each bar represents 100 air samples with a total of 1,200 data points represented in the figure. ACH, air changes per hour; MERV, minimum efficiency reporting value; PM, particulate matter.

Mean and median particle mass at PM 0.3 micron, PM 0.5 micron, PM 1.0 micron, PM 2.5 microns, PM 5.0 microns, and PM 10.0 microns were all significantly reduced by 77–100% (mean 88% reduction) with APC (*p* <0.0001) (Figures 2A,B).

In this real-world operating elevator cabin, APC reduced airborne particulate pollutants by significantly reducing particle mass for all particulate sizes measured. Reducing non-viable airborne particles is an excellent surrogate for reducing airborne pathogens (6). Thus, this indicates an ability to clear airborne pathogens as well. Of note is that the significant impact of APC was compared to a control with MERV 13 filter and 27 air changes each hour. ACTIVE Particle Control™ is effective on airborne particles of all sizes and very likely effective in clearing aerosolized bacteria and viruses. This work is consistent with other published studies (8, 9).

## Conclusion

In this first study of APC in an elevator cabin, the significant reductions in particle mass indicate that such an engineering modification may not only reduce exposure to particulate pollutants but could also reduce airborne transmission of viral or bacterial disease between elevator passengers.

## Data Availability Statement

The raw data supporting the conclusions of this article will be made available by the authors, without undue reservation.

## Author Contributions

ME: writing—original draft preparation (lead), writing—review and editing (lead), data curation (equal), visualization (lead), and formal analysis (lead). TW: investigation (equal), conceptualization (lead), and data curation (equal). MB: investigation (equal), methodology (lead), and data curation (equal). DH: resources (lead) and software (lead). All authors have read and agreed to the submitted version of the manuscript.

## Conflict of Interest

ME is the Chief Medical Officer and an investor in SecureAire, Inc., TW and MB are Mechanical Engineers at Henderson Building Solutions, LLC. DH is the Chief Technology Officer of and an investor in SecureAire, Inc. The experiments were conducted independently by Henderson Building Solutions, LLC.

## Publisher's Note

All claims expressed in this article are solely those of the authors and do not necessarily represent those of their affiliated organizations, or those of the publisher, the editors and the reviewers. Any product that may be evaluated in this article, or claim that may be made by its manufacturer, is not guaranteed or endorsed by the publisher.
